# *Solea senegalensis* sperm cryopreservation: New insights on sperm quality

**DOI:** 10.1371/journal.pone.0186542

**Published:** 2017-10-20

**Authors:** Marta F. Riesco, Catarina Oliveira, Florbela Soares, Paulo J. Gavaia, María T. Dinis, Elsa Cabrita

**Affiliations:** 1 CCMAR, University of Algarve, Campus of Gambelas, Faro, Portugal; 2 IPMA, IP, Olhão, Portugal; 3 DCBM, University of Algarve, Campus of Gambelas, Faro, Portugal; Institut National de la Recherche Agronomique, FRANCE

## Abstract

Cryopreservation of Senegalese sole sperm can represent an alternative to overcome some reproductive problems of this species. However, it is important to guarantee the safe use of cryopreserved sperm by selecting an appropriate protocol according to a high demand quality need to be ensured. It has been demonstrated that traditional assays such as motility and viability do not provide enough information to identify specific damage caused by cryopreservation process (freezing and thawing). Specific tests, including lipid peroxidation and DNA damage, should be performed. In the present study, motility and lipid peroxidation were performed as specific tests allowing us to discard cryopreservation conditions such as methanol as internal cryoprotectant and bovine serum albumin as external cryoprotectant. In addition, a caspase 3/7 detection by flow cytometry was performed to analyze apoptosis activity in the best selected conditions. Moreover, new highly sensitive tests based on transcript number detection have recently been described in fish sperm cryopreservation. For this reason, a transcript level detection assay was performed on certain oxidative and chaperone genes related to fertilization ability and embryo development (*hsp70*, *hsp90BB*, *hsp90AA*, *gpx*) to select the best cryopreservation conditions. DMSO+ egg yolk proved to be the best cryoprotectant combination in terms of transcript level. This study describes an optimized cryopreservation protocol for *Solea senegalensis* sperm demonstrating for the first time that transcript degradation is the most sensitive predictor of cell status in this species after cryopreservation.

## Introduction

Sperm cryopreservation has become a practice for artificial fertilization in species of commercial interest, endangered species or species with an interesting genotype. This technology could represent an important tool in Senegalese sole reproduction. In this species, low sperm volume and quality could be one of the causes of poor fertilization rates. Moreover, this species presents variable semen quality [1–4], thus reducing the chances of successful fertilization. Therefore, the possibility of sperm cryobanking would guarantee the continuous availability and gamete control quality. In Senegalese sole, cryopreserved sperm has traditionally been evaluated only by fertilization ability and the protocol used was directly applied from another flatfish species without any further assays [5,6]. In these studies, the successful fertilization rate was approximately 30% when cryopreserved sperm was used, which indicates the need to perform an exhaustive analysis to identify the type of damage that occurs. As in other species, it is well known that the cryopreservation process can damage spermatozoa at several levels such as plasma membrane (lipid peroxidation), mitochondria function and morphology [7,8]. This damage can ultimately affect cell viability and fertilization capacity. In addition, the importance of apoptosis activity detection in fish sperm, was revealed in a recent study, where authors implemented a sperm selection method for the recovery of a nonapoptotic sperm subpopulation. This study demonstrated that caspase detection in the identification of apoptotic cells in *S*. *senegalensis* seminal samples was more specific than other fluorescent dyes such as YO-PRO-1 [9]. YO-PRO1 is a green fluorescent DNA marker for cells with compromised plasma membrane acting as a potential early marker of cell death. Research on fish sperm has reported the use of this nucleic acid stain that permeate cells just after membrane destabilization, in conditions where the commonly used PI (propidium iodide) normally used with SYBR 14 does not permeate [9].

In view of this, specific tests are necessary to guarantee the total safety and efficiency of the cryopreservation process. Many efforts have recently been made in the study of DNA using different approaches such as the comet assay (single cell gel electrophoresis), TUNEL (terminal deoxynucleotidyl transferase-nick-end-labelling) and SCSA (sperm chromatin structure assay), since DNA damage may impair fertility or embryo development [10]. SCSA and TUNEL have not been used generally in fish sperm, however comet assay is widely employed which contributes to additional information on DNA damage, revealing the existence of single and double strand breaks in individual cells [11]. Recent studies have highlighted this type of damage in rainbow trout, European seabass, Senegalese sole and gilthead seabream sperm [12–16]. However, none of these techniques have been applied in Senegalese sole sperm cryopreservation until now. Although these specific tests provide a considerable number of data on cell status, they only give a general vision of chromatin damage and its integrity. In recent years, novel methods have been developed for the detection and quantification of relevant sperm transcripts based on qPCR, some of which have been used to evaluate genetic damage after cryopreservation in human [17] and other fish species such as gilthead seabream and zebrafish [18]. These assays provide information about different mRNA pattern profiles and can be used to detect different spermatozoa susceptibilities between species. Moreover, the studies in zebrafish and gilthead seabream sperm demonstrated that certain transcripts, such as *bdnf* and *kita*, were more abundant in good breeders with high fertility rates [18]. These studies provided a relevant observation showing that alterations in spermatozoa transcripts in fish have a direct effect on fertilization ability [18].

In this context, heat shock proteins (Hsps) are very relevant. Hsp are highly conserved molecular chaperones that participate in a wide spectrum of functions [19]. Moreover, in mammalian sperm, it has been demonstrated the critical role of *hsp70* and *hsp90* on fertilization ability [20,21] and post-fertilization events [22–24]. Matwee and colleagues [22], demonstrated that the exposure of mammalian embryos to antisense oligonucleotides complementary to *hsp70* mRNA not only significantly decreased development to the hatched embryos, but also increase the frequency of DNA fragmentation and apoptosis [23].

Other valuable indicators of oxidative stress and implicated on fertilization ability and embryo development have been described in human sperm [25]. Meseguer and colleagues [25] described the relationship between human sperm with low glutathione peroxidase (*gpx)* mRNA expression and cleavage asymmetry in the embryos. These results indicated that sperm derived transcripts conditioned embryo quality and persisted even to blastocyst stage [25].

Although different specific tests and candidate biomarkers have been used in sperm of several species, their application in Senegalese sole sperm cryopreservation has not been demonstrated until now. The aim of this study was to demonstrate the useful application of different specific tests and molecular tools for establishing an optimized sperm cryopreservation protocol in Senegalese sole. Therefore, these techniques would contribute to the knowledge of different types of damage and their consequences on fertilization success using cryopreserved sperm.

## Materials and methods

### Ethics statement

All experiments were performed according to directives PORT 1005/92 of the Portuguese direction for veterinary and food services (DGAV) and 2010/63/EU of the European Parliament and Council, and to guideline 86/609/EU of the European Union Council. CCMAR animal care committee was constituted after these experiments and therefore experimental approval by the institution was not necessary according to the Portuguese legislation. Nevertheless, all the persons involved in the experiments have a FELASA class C permit for animal experimentation, and the CCMAR facilities where the work was conducted are authorized by DGAV for animal experimentation. The authorization for experimental procedures with germ cells were previously approved by DGAV (ref.0421/000/000/2013).

### Animals

Senegalese sole wild breeders were acquired from Atlantik fish (privately owned land: 37°12'40.9"N 7°25'55.5"W9), sexed as described previously by Cabrita and colleagues [26], PIT-tagged and acclimated to captivity at the Ramalhete Experimental Station (37°0'27"N—7°59'41"W, CCMAR, Faro, Portugal) for at least one year prior to the experiments described hereinafter. Breeders were stocked into four 3 m^3^ indoor round fibreglass tanks, each containing 12 fish with a density of 5 kg/m^2^ (mean weight: 1.1±0.33 kg for males and 1.8±0.39 kg for females). The sex ratio was set at 2 to 1 (male:female). Water exchange was kept at 0.5 m^3^ /h and aeration was provided [2]. Photoperiod was simulated with a clock system according to environmental conditions in the area. Temperature varied according to external conditions maintaining thresholds of 21°C in spring-summer months (May-September) and 12°C in winter months (November-February). Broodstocks were fed on artificial pellets (SPAROS Lda.) at a daily ration of 1–3% biomass. According to the Directive 2010/63/UE on the protection of animals used for scientific purposes, the procedures described are classified as “mild” and no sacrifice of breeders was performed.

### Sperm collection

Sperm was collected from the 15^th^ of March to the end of June 2015 (corresponding to natural spawning season) when breeders were maintained at temperatures of 18±1.2°C. Individuals were anaesthetized with 300 ppm 2-phenoxyethanol during 10 min before sperm collection. Sperm collection was performed as previously described by Cabrita and colleagues [26]. The urogenital pore was dried, sperm was collected with a syringe or with a 20 μl micropipette by gently pressing the testes on the fish blind side. Samples were stored on ice in a Styrofoam support until further analysis, discarding samples contaminated with urine. Prior to the analysis, sperm was diluted 10-fold in a non-activating medium: Ringer solution (20 mM HEPES, 5 mM KH_**2**_PO_**4**_, 1 mM MgSO_**4**_, 1 mM CaCl_**2**_, 136 mM NaCl, 4.7 mM KCl, 300 mOsm/Kg, pH 7.5) and 5 μl of diluted sperm were used for measuring cell concentration and motility to discard low quality samples. Only pools with total motility higher than 75% were cryopreserved. Ten pools of sperm containing 10 males per pool were made (n = 10).

### Cryopreservation assays

*S*. *senegalensis* sperm was cryopreserved using the extender proposed for this species by Chereguini et al. [27] (Mounib solution: 125 mM sucrose, 100 mM KHCO_3_, 6.5 mM reduced glutathione). Different combinations of external and internal cryoprotectants (CPAs) were included. As internal CPAs, 10% dimethyl sulphoxide (DMSO), 10% dimethylformamide (DMF) and 10% methanol (MetOH) were tested. As external CPAs, 10% egg yolk (EY) and 1% bovine serum albumin (BSA) were added (S1 Fig: Graphical abstract). All chemicals were purchased from Sigma (Spain) and were of reagent grade or higher. Taking into account that the main objective of this work is to establish an optimized cryopreservation protocol in this species improving the previous protocol that contained 10% DMSO + 1% BSA, this combination was considered as the cryopreservation control media in this work.

A dilution rate of 1:2 in the extender (sperm: extender) was used according to the low sperm concentration found in this species. During 2 min of equilibration time, the sperm with the CPAs was loaded into 0.25 ml straws and after this time, straws were placed on a horizontal rack 2 cm above liquid nitrogen in a Styrofoam box. Sperm freezing was performed in nitrogen vapors for 10 min (S1 Fig: Graphical abstract). This freezing rate was described previously as the most suitable for this species by our group [6]. The straws were then immersed in liquid nitrogen and stored in a nitrogen container. Sperm samples were thawed in a water bath at 25°C for 10 s just before the beginning of sperm analysis. No washing step was performed after thawing to avoid the removing of the material released by the broken cells. The samples were processed immediately, for this purpose, 3 straws were thawed from the same treatment and pooled. All protocols were previously assayed in fresh sperm in order to discard CPA interference.

### Conventional sperm quality assays in Senegalese sole cryopreserved sperm

#### Motility

Total motility was determined in all samples using computer assisted sperm analysis (CASA) and ISAS software (ISAS, Proiser R+D, S.L., Spain). Motility analysis was performed activating 1 μl of post-thaw sperm with 5 μl of seawater (21°C and 35 ppt salinity). For all the analyses, motility was assessed in a Makler chamber using a phase-contrast microscope (Nikon 200, Japan) with a 10 x negative contrast objective and a digital camera (Basler A312f C-mount, Germany) set for 50 fps. The settings for CASA software were adapted for this species. Thawed sperm concentration was measured using the same software and the values ranged from 1 to 5 x 10^9^ spermatozoa/ml in all analyzed samples. The following CASA parameters were registered: percentage of motile cells and velocity according to the actual path (VCL; μm/s). Motility parameters were considered at 15, 30, 45 and 60 seconds post-activation. Both cell concentration and motility determination were repeated 3 times with 3 different subsamples. Experiments were performed in cryopreserved samples using 6–10 pools of 10 individual males for each treatment.

#### Viability

Propidium Iodide (PI-Sigma, Spain) was added at 1 μg/ml final concentration to detect dead cells. Immediately after, samples were acquired in a flow cytometer (FACSCalibur, BD Biosciences, CA, USA) adjusted for blue excitation (488 nm) line for the detection of PI (670/30). Flow cytometer settings were previously adjusted using a positive (100% dead cells) and a negative control (fresh sperm). Data analysis was performed applying Weasel 3.1 free software. A total of 75,000 events were counted for each sample. 8–10 pools of 10 individual males for each treatment were analyzed. The percentage of viable cells was recorded.

### Specific test development in Senegalese sole cryopreserved sperm

#### Comet assay

The comet assay was performed according to the method described by Cabrita et al. [28] with slight modifications. Briefly, 10 μl of semen were diluted in a non-activating solution (Ringer) to obtain a final concentration of approximately 10^6^ spermatozoa per ml. Cells were embedded in 0.5% agarose prepared in 0.1 M PBS and placed in agarose pre-coated slides. The slides were placed in a coplin jar containing the lysis solution (2.5 M NaCl, 100 mM Na_2_-EDTA, 10 mM Tris, 1% Triton X-100, 1% Lauril sarcosine, pH = 10) at 4°C for 1 h. For electrophoresis, the slides were placed in an electrophoresis cube (sub-Cell GT, Bio-Rad, Portugal) filled with approximately 1.5 l of electrophoresis solution (0.3 M NaOH, 1 mM Na_2_-EDTA, pH = 12). Electrophoresis was conducted at 25 V and 300 mA at 4°C for 10 min. After electrophoresis, the slides were drained and washed in the neutralizing solution (0.4 M Tris, pH = 7.5) at 4°C for 5 min (this step was performed twice). The slides were drained, fixed in pure methanol and stored at 4°C until further observation. After electrophoresis the slides were photographed (Nikon DS-Ri1, Japan) using PI (1 mg/mL) to stain DNA and observe the comets. A total of 100 cells were analyzed in the two slides performed by treatment. The percentage of DNA fragmentation (DNAt) was the parameter recorded with the KOMET 6.0 software (Andor Technology, Ireland). A total of 6–10 pools of 10 individual males for each treatment were analyzed.

#### Lipid peroxidation

To determine the lipid peroxidation level, the concentration of malondialdehyde (MDA) was quantified using a colorimetric assay (kit BIOXYTECH LPO-586^™^, OxisResearch^™^, USA), following the protocol described by Martínez-Páramo et al., [29], adapted for Senegalese sole sperm. MDA concentrations were calculated from a standard curve and presented as nmoles of MDA per million spermatozoa. Each cryopreserved sample (n = 6–10 pools) was processed in triplicate.

#### Caspase detection

In order to provide evidences on the cell status of cryopreserved sperm, the Caspase-3/7 assay using flow cytometry was carried out. For this purpose, thawed sperm from the two best CPAs conditions defined using the previous tests was stained with Muse Caspase-3/7 Kit (Millipore, Portugal) following the manufacturer’s instructions, using a positive and a negative control. This assay detects Caspase-3/7 activity binding to DEVD peptide substrate (this sequence is based on Caspase 3 and 7 cleavage sites). Moreover, a cell death dye, 7-aminoactinomycin D (7-ADD) was employed to detect changes in membrane permeability and providing information on membrane integrity. 7-AAD has been used to distinguish between viable cells (7-AAD negative) and apoptotic cells or dead cells (7-AAD bright) using the fact that permeability of the cell membrane, and hence fluorescence intensity, is low in early apoptotic cells and high in late apoptotic and dead cells. Immediately after, samples were acquired in a flow cytometer (FACSCalibur, BD Biosciences, CA, USA) equipped with a 488 nm laser for excitation of the Caspase 3/7 Green (FL2-H) and 7-ADD (FL3-H) fluorochromes and a 530/30 BP filter and a 690/50 BP filter for fluorescence emission. Data analysis was performed applying Weasel 3.1 free software. A total of 75,000 events were counted for each sample. For this analysis the best CPA conditions were analyzed (n = 3 pools with 10 individual males, and 3 technical replicates).

### Molecular characterization of Senegalese sole cryopreserved sperm

#### Gene expression

**RNA isolation and DNase treatment.** RNA was extracted from sperm cryopreserved using the best CPA combinations obtained in the previous tests (DMSO+EY and DMF+EY), using Tri-Reagent (Sigma, Spain) according to the manufacturer´s protocol adapted for fish sperm [18]. RNA quantity and purity were determined using a Nanodrop spectrometer (Nanodrop 1000, Thermo Scientific, Portugal). Total RNA isolated was treated with DNase using the DNase I, RNase free kit (Thermo Scientific, Portugal) for 30 min at 37°C to remove genomic DNA contamination. The quality of RNA samples was checked on an agarose gel. The isolated RNA showed high purity (A260/280 > 1.8) and was stored at -80°C until further use.

**Reverse transcription.** Complementary DNA (cDNA) was obtained from 1 μg RNA using the cDNA synthesis kit (Bio-Rad, Portugal), following the manufacturer´s protocol. The cDNA from cryopreserved sperm with the best CPA conditions (DMSO+EY and DMF+EY) was stored at -20°C before qPCR analysis. Reverse transcription (RT-PCR) conditions were 25°C for 5 min, 42°C for 30 min and 85°C for 10 min.

**Real-time PCR analysis.** Real time PCR analysis was carried out using an iCycler Q Multicolor PCR Detection system (Bio-Rad, Portugal). Reactions were performed in 20 μl volume containing 10 μl of SsoFast EvaGreen Supermix (Bio-Rad, Portugal), 2 μl of primers (0.5 mM) (Table 1) and 2 μl of cDNA template. The amplification protocol used was as follows: initial denaturation for 10 min at 95°C, followed by 40 cycles of 95°C for 15 s and 60°C for 1 min, and finally 5 s at 95°C. The primers and amplification protocols chosen for expression assays were as previously published for *hsp70* [30], glutathione peroxidase (*gpx)* [30], *hsp90AA* and *hsp90BB* [31], respectively (S1 Fig: Graphical abstract and Table 1). For *gpx* the protocol was the same as previously reported, but the annealing temperature was modified to 63°C. All these transcripts were selected for their implication on fertilization ability and post-fertilization events. Expression levels of each Senegalese sole transcript relative to the ubiquitin housekeeping gene (*ubq*) were calculated for all transcripts using the delta-delta-Ct (2^-ΔΔCt^) method, which is an algorithm to analyse relative changes in gene expression. It requires the assignment of one housekeeping gene, which is assumed to be uniformly and constantly expressed in all samples [32]. Results were expressed as the mean ± SE of the 2^-ΔΔCt^ method of three independent experiments of three different pools of males containing ten individual males. DMSO+EY was used as reference treatment.

**Table 1:** List of primers for *S*. *senegalenesis* transcript abundance assays. Gene name, abbreviation, sequences start from 5´ to 3´, reference, annealing temperature and amplicon size are specified for each pair of primers.

GENE	REFERENCE	ABBREVIATION	OLIGO SEQUENCE 5´-3	TM (°C)	AMPLICOM LENGHT (BP)
Heat shock protein 70 kDa protein	[30]	*hsp70*	F-GCTATACCAGGGAGGGATGGAAGGAGGG	60	119
			R-CGACCTCCTCAATATTTGGGCCAGCA		
Heat shock protein 90 kDa protein cDNA variant AA	[31]	*hsp90AA*	F-GACCAAGCCTATCTGGACCCGCAAC	60	105
			R-TTGACAGCCAGGTGGTCCTCCCAGT		
Heat shock protein 90 kDa protein cDNA variant BB	[31]	*hsp90BB*	F-TCAGTTTGGTGTGGGTTTCTACTCGGCTTA	60	148
			R-GCCAAGGGGCTCACCTGTGTCG		
Glutathione peroxidase	[30]	*gpx*	F-GATTCGTTCCAAACTTCCTGCTA	63	212
			R-GCTCCCAGAACAGCCTGTTG		
β-actin	[33]	*actb2*	F-AATCGTGACCTCTGCTTCCCCCTGT	60	114
			R-TCTGGCACCCCATGTTACCCCATC		
Translation initiation factor eIF-α subunit	[33]	*ef1α*	F-GATTGACCGTCGTTCTGGCAAGAAGC	60	142
			R-GGCAAAGCGACCAAGGGGAGCAT		
Glyceraldehyde-3-phosphate dehydrogenase	[33]	gapdh2	F-AGCCACCGTGTCGCCGACCT	60	107
			R-AAAAGAGGAGATGGTGGGGGGTGGT		
18s RNA	[33]	*18s*	F-GAATTGACGGAAGGGCACCACCAG	60	148
			R-ACTAAGAACGGCCATGCACCACCAC		
Ubiquitin	[33]	*ubq*	F-AGCTGGCCCAGAAATATAACTGCGACA	60	93
			R-ACTTCTTCTTGCGGCAGTTGACAGCAC		

**Housekeeping gene stability study.** Taking into account that the stability and uniformity of housekeeping gene is crucial in the 2^-ΔΔCt^ method, we carried out a previous analysis regarding reference gene stability according to published studies in Senegalese sole larvae [33]. For this study, we analysed highly expressed genes such as glyceraldehyde-3-phosphate dehydrogenase (*gapdh2*), β-actin (*actb2*), 18S ribosomal (*18s*), ubiquitin *(ubq)* and subunit a1 of the elongation factor 1 gene (*ef1α)* that have been considered as putative housekeeping genes in Senegalese sole larvae (Table 1) [33]. The stability of these housekeeping genes were previously analysed using the different tested cryoprotectants (DMSO and DMF) and fresh sperm samples in order to demonstrate their stability in a specific physiological process (cryopreservation) or condition (using DMSO or DMFA). For this analysis the best CPA conditions were analysed comparing to fresh sperm samples (n = 3 pools with 10 individual males, and 3 technical replicates) (S2 Fig). NormFinder software was also used to evaluate the candidate normalization genes. NormFinder has the ability to measure the gene expression stability taking into account intra- and inter-variations in defined sample groups or treatments. The combination of these estimates provides a direct measure of the variation in expression for each gene. This function determinates the best reference genes for normalization [34].

### Statistical analysis

Data were analyzed using SPSS V.22 (IBM, USA) and Microsoft Excel. To analyse the effects of different cryopreservation protocols on sperm motility, a general linear model with the Bonferroni correction was used (p< 0.05). One-way ANOVA (p<0.05) was performed followed by Student-Newman-Keuls (S-N-K) as a post hoc test for viability, comet assay and lipid peroxidation results after Senegalese sole sperm cryopreservation. Results were expressed as mean of percentages in all analyses of 6–10 pools containing 10 individual males. A Principal Component Analysis was performed to reduce the dimensionality of a data set in which there are a large number of interrelated variables, while retaining as much as possible of the variation present in the data set (S3 Fig). In caspase activity studies, the Student´s t-test between the two best CPA conditions was also performed. Results were expressed as mean of percentages of 3 pools of 10 individual males.

qPCR results were expressed as the mean ± SE of the 2^-ΔΔCt^ method. The Student´s t-test (μ = 1) was performed according to previous studies [35] to identify changes in transcript levels between both cryopreservation treatments (DMSO+EY and DMF+EY).

## Results

### Conventional sperm quality assays in Senegalese sole cryopreserved sperm

#### Motility

**Fig 1:**
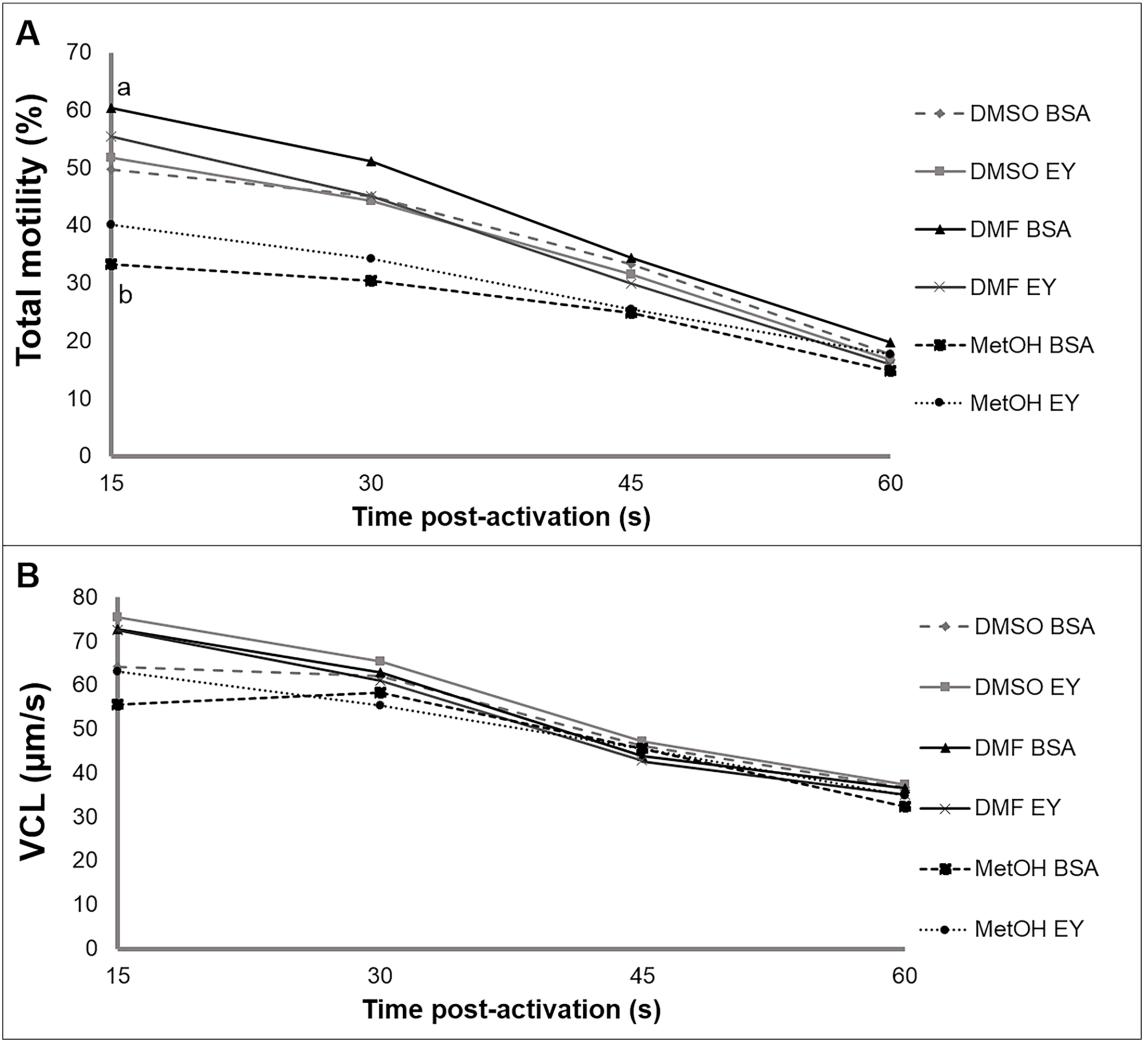
*S*. *senegalensis* sperm motility after cryopreservation. Three different internal cryoprotectants (CPAs) were tested: dimethyl sulfoxide (DMSO), dimethyl formamide (DMF) and methanol (MetOH), at 10% concentration, supplemented with two different external CPAs: 1% bovine albumin serum (BSA) and 10% egg yolk (EY). The following CASA parameters were recorded: **(A)** percentage of motile cells and **(B)** velocity according to the actual path (VCL; μm/s). Motility parameters were considered at 15, 30, 45 and 60 seconds post-activation. All results were expressed as a mean of percentages ± SE of 6–10 pools of 10 individual males for each treatment. A general linear model with the Bonferroni correction was used (p< 0.05). Different letters show significant differences between conditions (p<0.05).

In general, cryopreserved sperm with different CPA combinations showed high initial motility (15 s post activation) (Fig 1A). In the case of samples cryopreserved with MetOH, regardless of the external CPA employed (10% EY or 1% BSA), the percentages of total sperm motility (15 s post activation) were significantly lower than 40% (p<0.05). When these samples were compared to samples cryopreserved with the other cryoprotectants with both external CPAs, DMSO or DMF presented higher motility (50–60%). Sperm motility decreased with time post-activation (from 15 to 60 s) as we expected, showing the same pattern among the different cryopreservation protocols (Fig 1A). MetOH+BSA samples showed significant lower motility percentages (p<0.05) but a lesser decrease in motility over time in comparison with those cryopreserved with 10% DMF+BSA (Fig 1A).

Regarding sperm curvilinear velocity (VCL), no significant differences were found among CPAs. General values were higher than 55% in all the studied treatments (Fig 1B).

#### Viability

**Fig 2:**
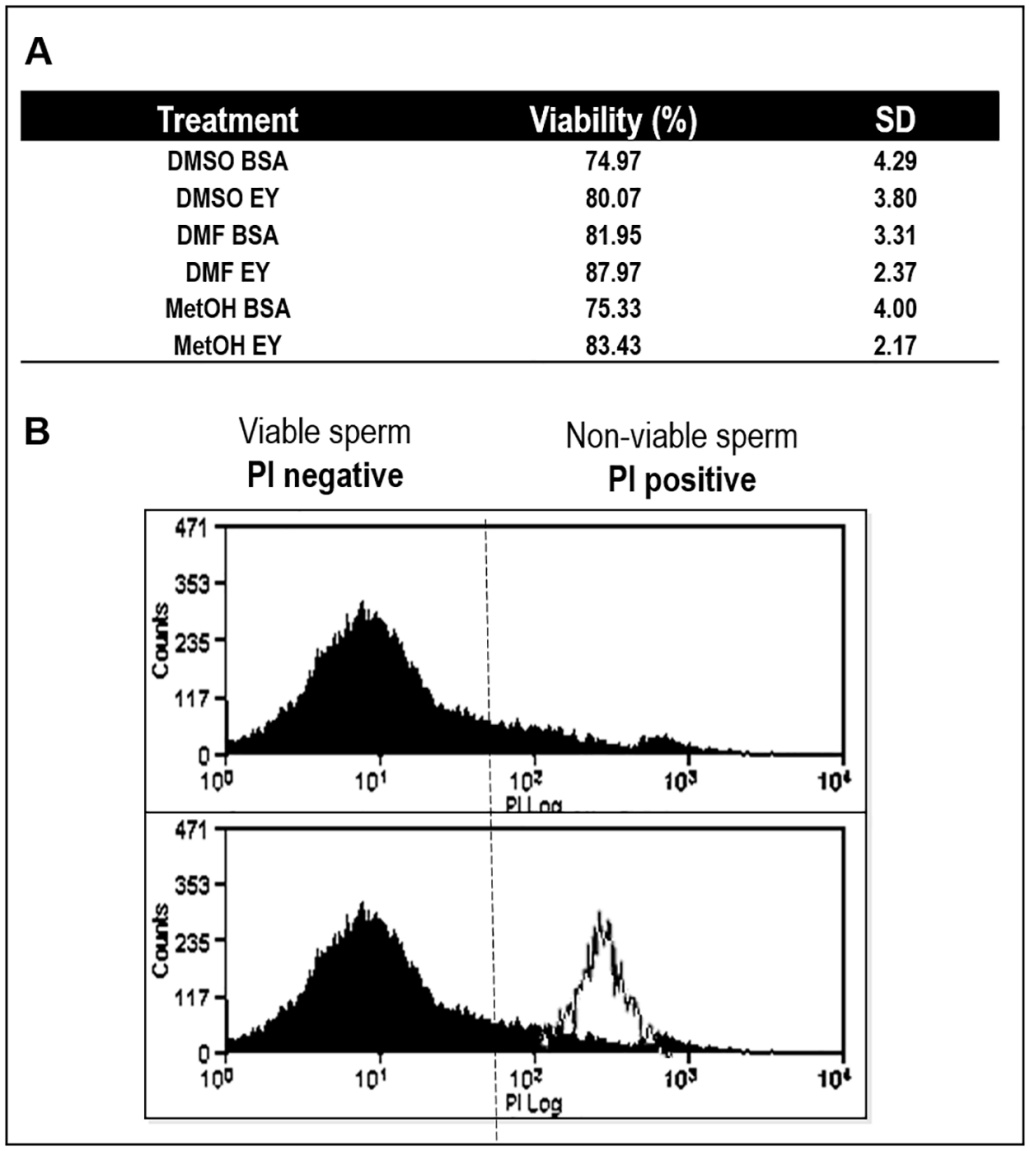
*S*. *senegalensis* sperm viability after cryopreservation. A) Three different internal cryoprotectants (CPAs) were tested: dimethyl sulfoxide (DMSO), dimethyl formamide (DMF) and methanol (MetOH), at 10% concentration, supplemented with two different external CPAs: 1% bovine albumin serum (BSA) and 10% egg yolk (EY). The percentage of viable cells was expressed as a mean of percentages ± SE of 8–10 pools of 10 individual males for each treatment. One-way ANOVA (p<0.05) was performed and no significant (p< 0.05) differences were found among the cryopreservation media. B) Flow cytometry histograms obtained after propidium iodide (PI) staining.

Viability results obtained by PI staining analysed by flow cytometry showed no significant differences in the percentage of viable cells among the different cryopreservation protocols. All viability percentages were higher than 70%, resulting in high viability rates after cryopreservation (Fig 2).

Linking the motility and viability results, 10% MetOH supplemented with 10% EY or 1% BSA as external cryoprotectants were the most toxic combinations for Senegalese sole spermatozoa. Although no significant differences were observed in the viability parameter, MetOH obtained the lowest percentage of viable cells when combined with BSA (Fig 2).

### Specific test development in cryopreserved Senegalese sole sperm

#### Comet assay

**Fig 3:**
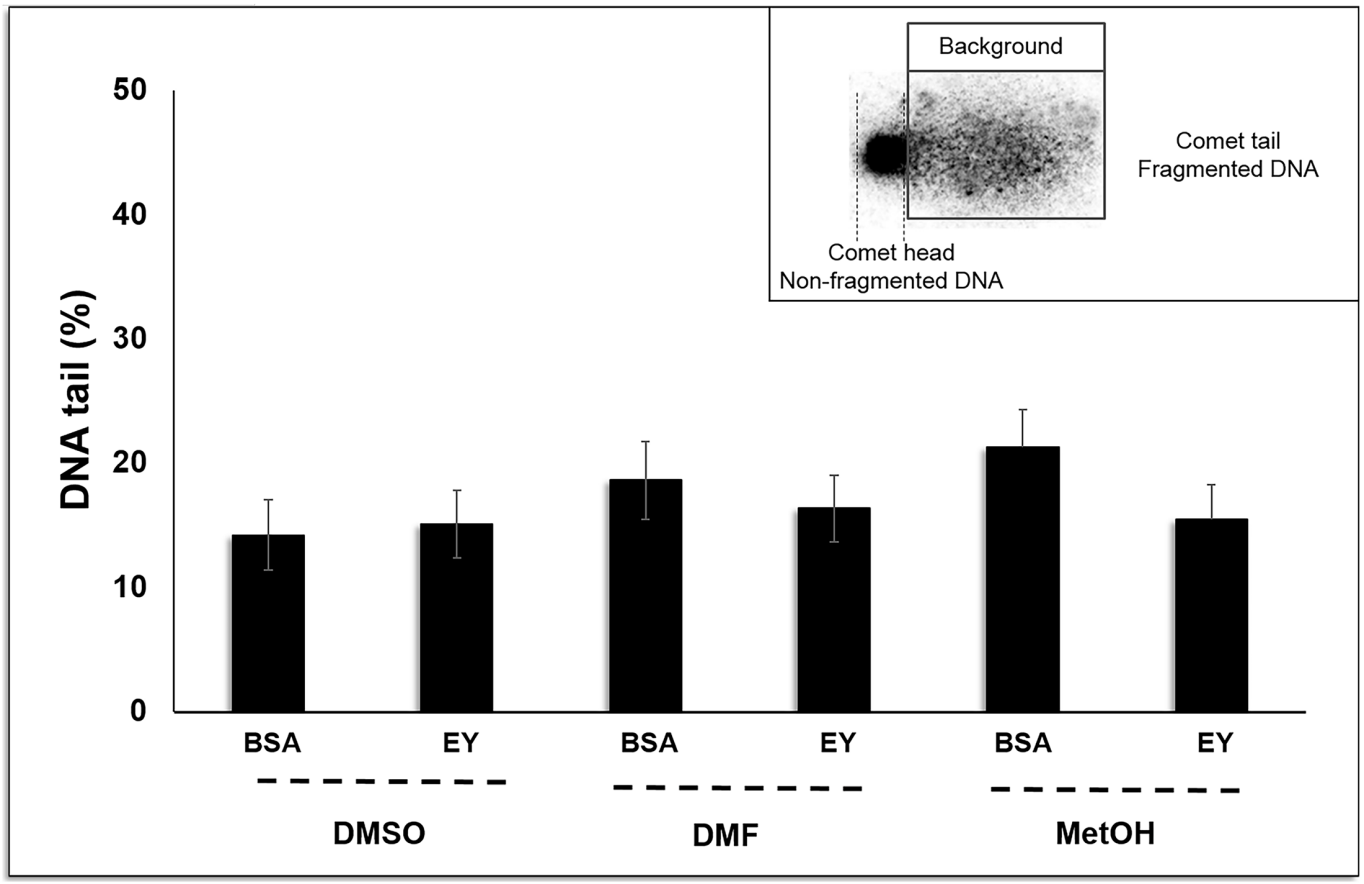
*S*. *senegalensis* sperm fragmentation after cryopreservation. Three different internal cryoprotectants (CPAs) were tested: dimethyl sulfoxide (DMSO), dimethyl formamide (DMF) and methanol (MetOH), at 10% concentration, supplemented with two different external CPAs: 1% bovine albumin serum (BSA) and 10% egg yolk (EY). The percentage of fragmented DNA (DNAt) was expressed as a mean of percentages ± SE of 6–10 pools of 10 individual males for each treatment. One-way ANOVA (p<0.05) was performed and no significant (p< 0.05) differences were found among the cryopreservation media. A total of 100 cells were analyzed in the two slides performed by treatment.

When DNA fragmentation was analysed in cryopreserved samples using different CPA combinations, a low percentage of DNA damage was found after thawing (Fig 3). All obtained fragmentation rates provided as percentage of DNA in the tail (DNAt) were lower than 20% except in the samples cryopreserved with MetOH+BSA. There were no significant differences among treatments and the highest percentage of DNA fragmentation was found in the samples cryopreserved with MetOH+BSA (21.28%) (Fig 3).

#### Lipid peroxidation

**Fig 4:**
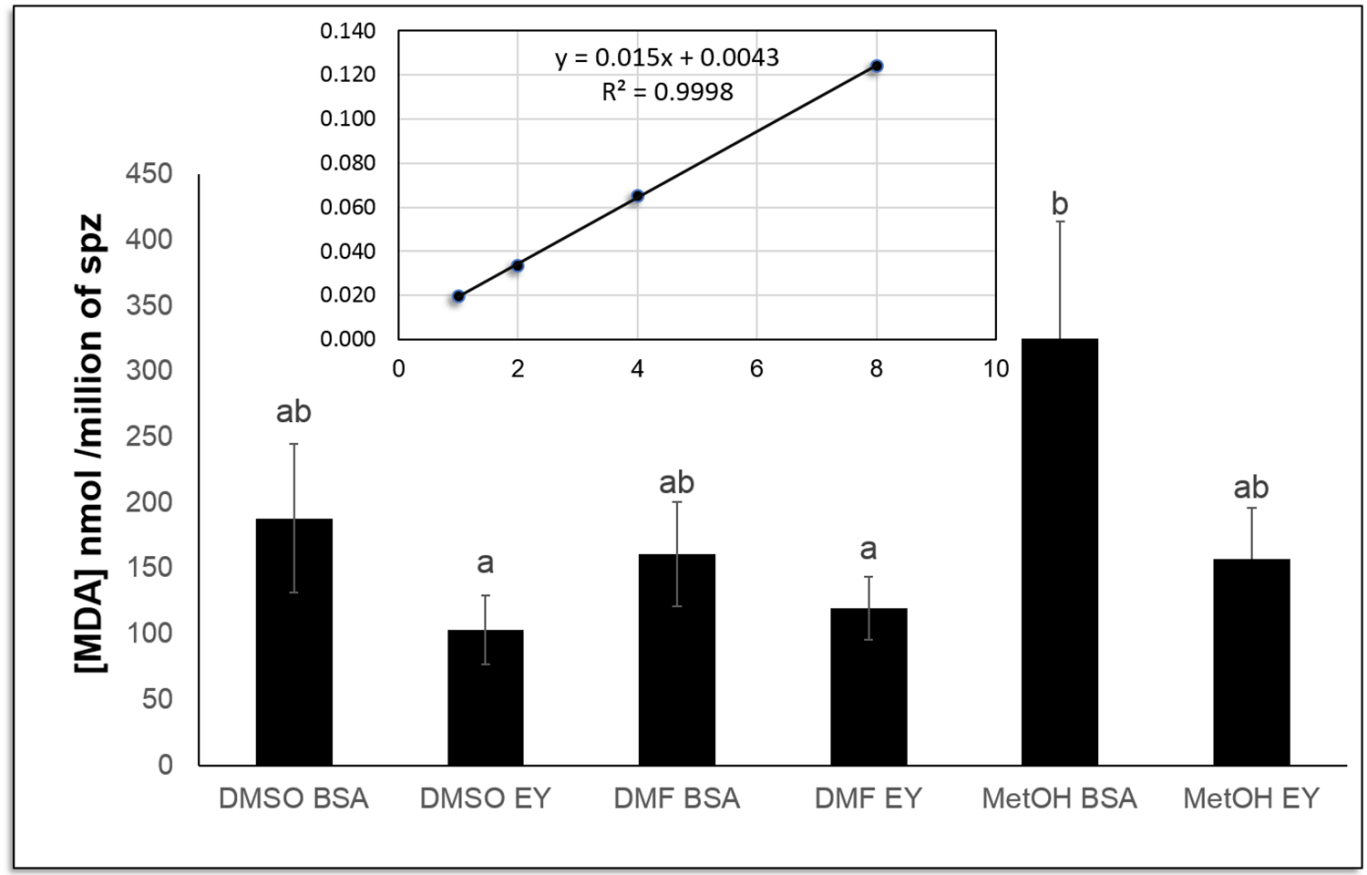
*S*. *senegalensis* sperm lipid peroxidation after cryopreservation. Three different internal cryoprotectants (CPAs) were tested: dimethyl sulfoxide (DMSO), dimethyl formamide (DMF) and methanol (MetOH), at 10% concentration, supplemented with two different external CPAs: 1% bovine albumin serum (BSA) and 10% egg yolk (EY). The concentration of malondialdehyde (MDA) presented as nmoles of MDA per million spermatozoa were expressed as a mean ± SE of 6–10 pools of 10 individual males for each treatment. One-way ANOVA (p<0.05) was performed followed by Student-Newman-Keuls (S-N-K) as a post hoc test (p< 0.05). Different letters show significant differences between conditions (p<0.05).

Malondialdehyde (MDA) was determined in all cryopreserved samples as a measure of lipid peroxidation status. The highest MDA concentration was found in sperm samples cryopreserved with 10% MetOH+BSA (325.14 nmoles of MDA per million spermatozoa) (Fig 4). In contrast, the lowest lipid peroxidation levels were found in samples cryopreserved with DMSO or DMF when EY was used as external CPA (103.69 and 120.36 nmoles of MDA per million spermatozoa, respectively) (Fig 4). These combinations showed a significant (p<0.05) lower MDA concentration resulting in an improvement in lipid peroxidation protection.

A Principal Component Analysis was performed to confirm the best sperm quality descriptor when comparing cryopreserved samples. According to PCA results, two components were obtained, one explaining 93% of the total variance of our results (component 1), and the other explaining 5% (component 2). Component 1 corresponds to lipid peroxidation data, and component 2 corresponds to motility parameters. In view of these results, all treatments containing MetOH and BSA were discarded in the next step of the cryopreservation analysis (S3 Fig).

#### Caspase detection

**Fig 5:**
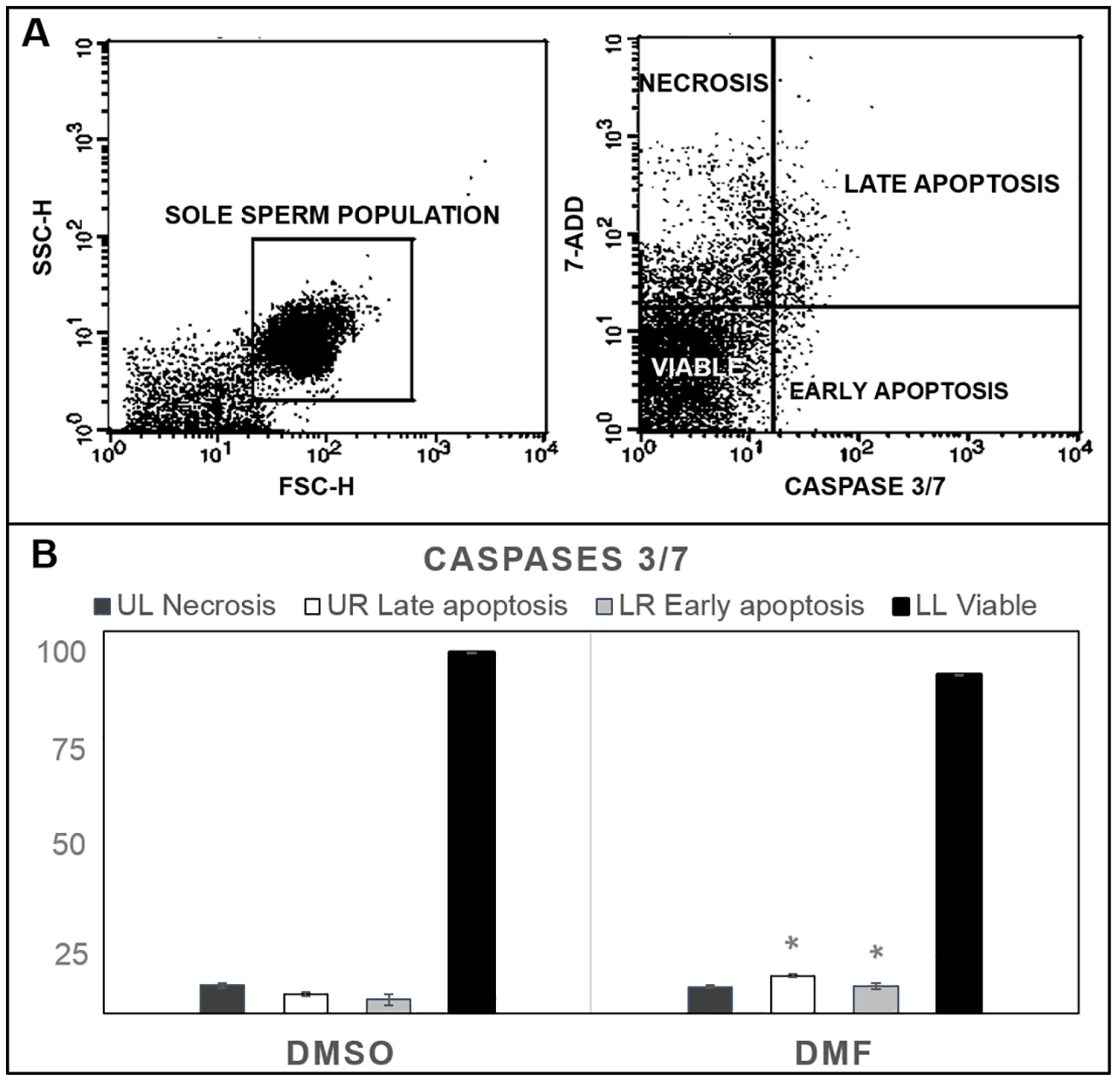
*S*. *senegalensis* sperm caspase detection after cryopreservation. Caspase detection by flow cytometry under the best CPA conditions: dimethyl sulfoxide (DMSO) dimethyl formamide (DMF) supplemented with 10% egg yolk (EY).Thawed sperm was stained with Caspase 3/7 Green (FL2-H) and 7-ADD (FL3-H) fluorochromes (530/30 BP and a 690/50 BP fluorescence emission filters). The percentages of early apoptosis, late apoptosis, necrosis and viable cells were expressed as the means ± SE of three independent experiments with 3 pools of 10 individual males for each. The Student’s t-test between the two best CPA conditions was performed. Asterisks show significant (p<0.05) differences between treatments.

A detection of different caspases involved in early and late apoptosis was carried out under the best selected CPA conditions (DMSO+EY and DMF+EY) in Senegalese sole sperm. The percentage of early and late apoptotic cells between both cryopreservation conditions was different, revealing a significant increase (p<0.05) in sperm samples cryopreserved with DMF+EY (6.4% early apoptosis and 8.9% late-apoptosis) in comparison with DMSO+EY (3.3% early apoptosis and 4.6% late apoptosis) (Fig 5). No significant differences were found in viable cells between cryopreservation treatments, which are in accordance with previous data obtained in viability studies using PI staining and flow cytometry (Fig 1).

### Molecular characterization of Senegalese sole cryopreserved sperm

#### Housekeeping gene stability

Taking into account that housekeeping gene stability is the most important parameter for accurate normalization in gene expression studies, a housekeeping gene stability expression study was performed. *ubq* proved to be the most suitable reference gene in terms of stability according to previous studies performed in *S*. *senegalensis* larvae and to our own results [33]. Ct values varied between 15 (*18s* rRNA) and 26.14 (*gapdh2)*. *actb2*, *gpdh2 a*nd *ef1α* were expressed at low levels in *S*. *senegalensis* sperm (median Ct values around 23 cycles). The smallest Ct variation was exhibited by *ubq* (0.606 stability value) housekeeping gene in Senegalese sole comparing fresh sperm samples with different cryopreserved ones (DMSO+EY or DMF+EY) (S2 Fig).

On the contrary, *gapdh2* (0.746), *actb2* (0.960), *ef1α* (0.691) and *18s* (1.814) showed the most variable expression levels corresponding to higher stability values in *S*. *senegalensis* sperm by NormFinder software analysis, which is in accordance to previously studies published in Senegalese sole larvae [33] (S2 Fig).

#### Gene expression assay

**Fig 6:**
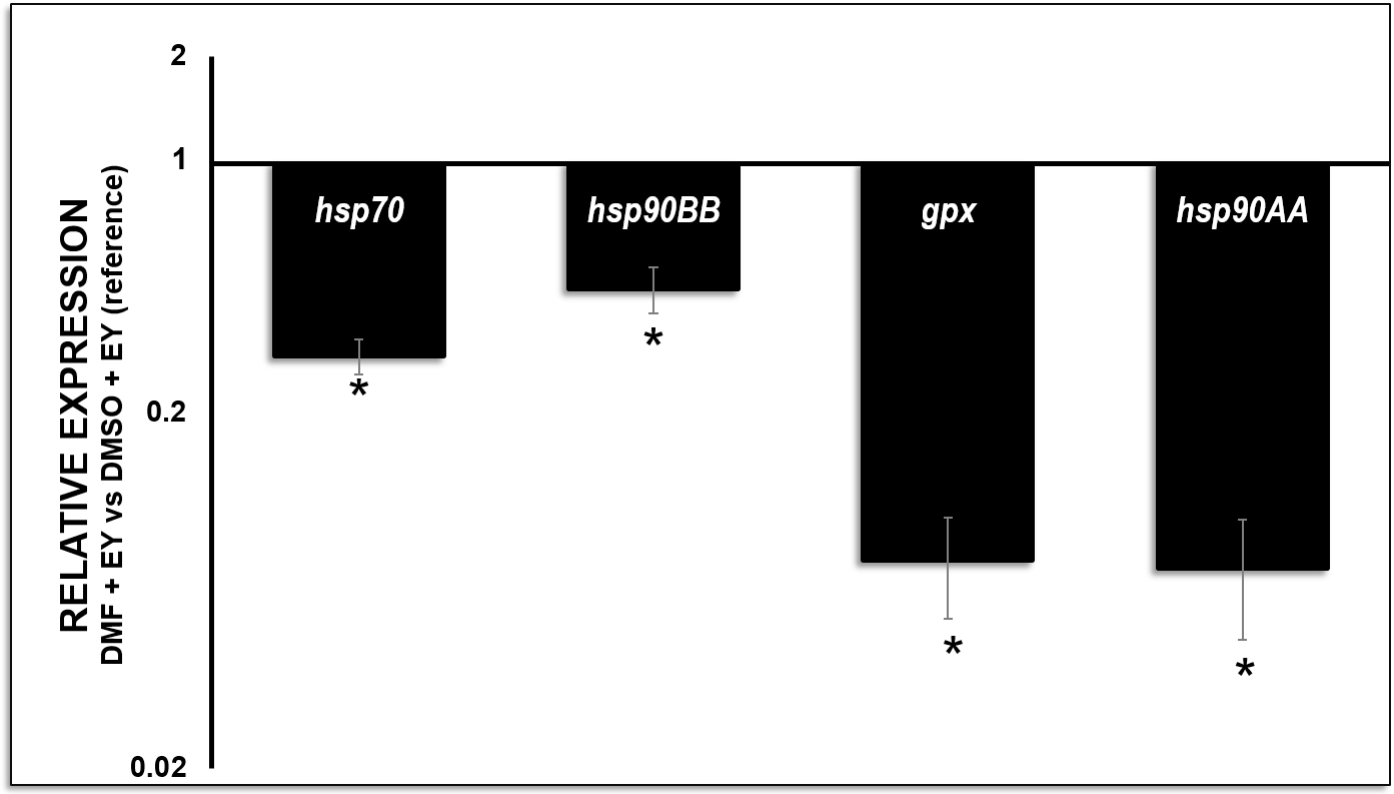
Expression levels of *S*. *senegalensis* sperm transcripts. Abundance of Senegalese sole transcripts after cryopreservation with the best CPA conditions: dimethyl formamide (DMF) supplemented with 10% egg yolk (EY) using as reference treatment dimethyl sulfoxide (DMSO) with 10% egg yolk (EY). Transcript levels for each gene relative to *ubq* were calculated for all samples using the 2−^ΔΔCt^ method. All results were expressed as the means ± SE of three independent experiments with three replicates each. The Student’s t-test (μ = 1) was performed to identify changes in transcript levels after cryopreservation. Asterisks show a significant (p<0.05) difference in transcript level.

Transcript level characterization in DMF+EY cryopreserved sperm showed a significant decrease (p<0.05) in all studied transcripts when comparing to DMSO+EY used as reference treatment for this study (Fig 6). This decrease was higher in the *gpx* and *hsp90AA* transcripts where the differences between both treatments were higher than ten-fold decrease in DMF+EY treatment comparing to DMSO+EY as reference treatment. In the case of *hsp90BB*, previously described as less sensitive to thermal variations in this species [31], we observed the lowest significant decrease (p<0.05) in transcript level in the case of DMF+EY (0.44 fold) in comparison with the DMSO+EY (Fig 6).

## Discussion

Sperm cryopreservation is an important tool for reproductive management in Senegalese sole. The total safety of the technique should be guaranteed in order to obtain an optimized and standardized cryopreservation protocol. It is therefore necessary to identify the type of damage caused by cryopreservation, by performing specific tests at different levels. Some of these assays were previously carried out in cryopreserved sperm of other important fish species as gilthead seabream [10], but not in post-thaw sperm from Senegalese sole. In fact, this is the first study that attempts to analyse post-thaw sperm quality in this species. The importance of traditional tests such as motility and viability are evident and widely demonstrated in all sperm cryopreservation protocols [29,36]. These assays are the first indicators in sperm quality after thawing in most species. In our study, samples cryopreserved with MetOH, regardless of the external CPA employed (10% EY or 1% BSA), registered the lowest percentages of total sperm motility when these samples were compared to samples cryopreserved with the other cryoprotectant media. In the case of viability results, no differences were found among the different cryopreservation media. However, these tests cannot allow differentiating certain types of damage, and therefore some detrimental conditions cannot be discarded. This is especially important in species where cryopreserved sperm seems to be less prone to suffer damage compared with other species that cryodamage is more evident (e.g. European seabass) [29].

The second line of analysis consists on specific tests performed on cell damage such as lipid peroxidation, DNA status and apoptosis. Oxidative stress is an important factor, which influences the production of seminal Reactive Oxygen Species (ROS); these ROS can cause sperm dysfunction through lipid peroxidation of the sperm membrane and a decrease in fertilization ability [37]. Moreover, several studies demonstrated that ROS attacks the chromatin integrity causing nucleotide modifications, DNA strand breaks and cross-linking [38]. In addition, production of ROS, which can damage DNA, proteins, and lipids [39], can ultimately lead to apoptosis or necrosis in living cells [40]. In our study, lipid peroxidation assay played an important role in discarding MetOH treatment and in establishing the best external CPA, egg yolk (Figs 1A and 4). According to previous studies in European sea bass, lipid peroxidation has an important role in sperm quality as it can be considered an important and consistent marker [29]. Concerning to DNA fragmentation, no significant differences were found among the cryopreservation media. The obtained fragmentation percentages in cryopreserved sperm were lower (<25%) compared to data obtained by other authors for marine species such as European seabass, 38.2% DNAt, [41], or gilthead seabream, 28.23%, for DNAt [28].

The assessment of seminal oxidative stress together with traditional sperm parameters may play an important role in the diagnosis of sperm quality, providing an exhaustive perspective of the consequences of the cryopreservation process and determining different susceptibilities to several types of damage [37].

In our study, these assays contributed to selecting and discarding some CPA conditions in Senegalese sole sperm cryopreservation demonstrating that MetOH showed the most toxic effect on cells in terms of lipid peroxidation and sperm motility. Statistical analysis using PCA confirmed these findings. The detrimental effect of MetOH on sperm motility has been previously reported in zebrafish sperm [42].

In terms of the effects of cryopreservation on spermatozoa apoptosis, we performed caspase 3/7 detection by flow cytometry in the best obtained CPA conditions (DMSO+EY and DMFA+EY), since the activation of these caspases is sufficient to induce apoptosis [43]. We observed a significant increase in apoptotic cells in Senegalese sole sperm cryopreserved with DMF+EY in comparison with DMSO+EY (Fig 5), detecting events related with early and late apoptosis. Apoptotic sperm should be avoided in order to increase the opportunities of successful fertilization as previously demonstrated in sperm cryopreservation [44,45].

In recent years, novel molecular methods for germ cells quality characterization have been described [46]. These techniques are based on the detection and quantification of some important transcripts in sperm, critical to fertilization and successful embryo development [17,24,47]. It is assumed that mRNA molecules present in spermatozoa are remnants of the transcripts from spermatogenesis [48], playing a specific role in the cell [17,24,47,49]. In previous studies in human sperm it has been found that freezing and thawing process produce an alteration of certain transcripts [17], and it might represent an important tool for validating particular cryopreservation protocols [36]. However, the alteration in the number of sperm transcripts caused by cryopreservation only can be explained by a degradation process since spermatozoa is a transcriptionally inactive cell [50]. In addition, there are some sperm genes that present a higher susceptibility to cryo-damage depending on their chromosomal localization and the structural organization of the chromatin [51,52] as previously demonstrated in zebrafish germ cells [46,53], trout [52] and human sperm [17].

In this work, qPCR results proved to be an effective assay as it enabled to differentiate between two of the best cryopreservation treatments. The selected transcripts (*hsp70*, *hsp90* and *gpx*) are involved in sperm quality [19,20], fertilization and successful embryo development [20–23] in mice and humans. *gpx* transcript degradation in human sperm cells was correlated to the presence of asymmetric embryos indicating that sperm-derived mRNA may condition human embryo quality and persist even to blastocyst stage [25]. Moreover, the quantity of *gpx* mRNA has been correlated to post-thaw sperm motility in humans [47]. Therefore, this mRNA can be employed as indicators of post-thaw sperm quality [47]. Taking into account our results, where a significant decrease in *gpx* transcript was observed in sperm samples cryopreserved with DMF+EY, and according to previous evidences in human sperm, we hypothesised that the use of these samples in fertilization assays would compromise the normal embryo development in Senegalese sole.

In the case of *hsp70* transcript, the presence of mRNA in spermatozoa may also play a role in post-fertilization events, this chaperone may be required as a stress protector during early cleavage prior to the activation of the embryonic genome [23]. Moreover, the inducible form *hsp90AA* has been reported as a useful marker in Senegalese sole larvae response to thermal stress caused by high temperature, contrary to the *hsp90BB* cDNA variant [31]. The differences found in transcript degradation after cryopreservation between the best selected conditions could be crucial, taking into account the participation of these transcripts in fertilization success and the correct early embryonic development. Therefore, although these transcripts are present in maternal genome [54,55], it seems that their presence in the sperm is crucial prior to the activation of the embryonic genome [25]. In our study, we observed a significant increase in degradation of all studied transcripts in DMF+EY treatment respect to DMSO+EY (reference treatment, Fig 6). In the case of *hsp90*, we analysed two published cDNAs coding Senegalese sole *hsp90* (*hsp90AA* and *hsp90BB* transcripts). Our results demonstrated that *hsp90AA* is a more sensitive marker in response to thermal conditions in Senegalese sole than *hsp90BB*, described previously by other authors as less suitable in this thermal response [31]. This transcript degradation points out, again, that DMSO+EY gives better protection to the cells at low temperatures, preventing losses of these crucial transcripts.

Taking into account all the obtained results, DMSO+EY provides the best conditions in Senegalese sole sperm cryopreservation when compared with other cryoprotectant media. It is generally known that DMSO is the most widely used cryoprotectant for the cryopreservation of fish sperm and provides better protection [56]. Moreover, the use of egg yolk (EY) as external cryoprotectant has been reported to cover and interact with the cell membrane due to their low-density lipoprotein fraction thereby reducing lysis during the freezing process [57,58]. However, the beneficial effects of EY supplementation appear to be species specific as increased fertilization rates have been observed in Atlantic salmon [59], rainbow trout [60] and common carp [61] but not in other fish species as northern pike [62] or asp [63].

Molecular tools are an important and useful step for selecting the most suitable cryopreservation protocol and its standardization, ensuring the total safety of this technique. This study represents the first step towards the possibility of selecting cryopreservation protocols in this species from a molecular point of view, demonstrating that transcript degradation is the most sensitive predictor of cell status. Further studies are being performed in order to demonstrate the real consequences of this specific transcript degradation on fertilization ability.

## Supporting information

S1 Fig Graphical abstract: *S*. *senegalensis* sperm cryopreservation protocol and the different assays performed after thawing. **A)** Sperm cryopreservation protocol, **B)** Conventional assays, **C)** Specific assays, **D)** Novel and molecular assays.
(TIF)

S2 Fig *S*. *senegalensis* sperm housekeeping gene stability studies. Different housekeeping genes were selected as glyceraldehyde-3-phosphate dehydrogenase (*gapdh2*), β-actin (*actb2*) or 18S ribosomal (*18s*) RNA, ubiquitin (*ubq*), and subunit a1 of elongation factor 1 gene, (*ef1α*). The Ct value indicates the fractional cycle at which fluorescence intensity reached the fluorescence threshold. For this analysis the best CPA conditions were analysed: dimethyl sulfoxide (DMSO) and dimethyl formamide (DMF) supplemented with 10% egg yolk (EY), compared to fresh control. Transcriptional levels (Ct values) of candidate reference genes were expressed as a mean ± SE (n = 3, and 3 technique replicates). The stability value was calculated after a 2^^-Ct^ transformation using Normfinder software.
(TIF)

S3 Fig *S*. *senegalensis* sperm quality analysis analysed by a Principal Component Analysis (PCA). A Principal Component Analysis was performed to reduce the dimensionality of a data set. PCA results defined two components, one explaining 93% of the total variance of our results (component 1), and the other explaining 5% (component 2). Component 1 corresponds to lipid peroxidation data, and component 2 corresponds to motility parameters.
(TIF)

## References

[1] Beirão J, Soares F, Herráez MP, Dinis MT, Cabrita E. Changes in *Solea senegalensis* sperm quality throughout the year. Anim Reprod Sci. 2011;126: 122–129. doi: 10.1016/j.anireprosci.2011.04.009 21571455 10.1016/j.anireprosci.2011.04.009

[2] Beirão J, Soares F, Herráez MP, Dinis MT, Cabrita E, Cabrita E, et al Sperm quality evaluation in *Solea senegalensis* during the reproductive season at cellular level. Theriogenology. Elsevier; 2009;72: 1251–1261. 10.1016/j.theriogenology.2009.07.021 19781754

[3] Cabrita E, Soares F, Dinis MT. Characterization of Senegalese sole, *Solea senegalensis*, male broodstock in terms of sperm production and quality. Aquaculture. 2006;261: 967–975.

[4] Martinez-Pastor F, Cabrita E, Soares F, Anel L, Dinis MT. Multivariate cluster analysis to study motility activation of *Solea senegalensis* spermatozoa: a model for marine teleosts. Reproduction. Society for Reproduction and Fertility. 2008;135: 449–459. 10.1530/REP-07-0376 18367506

[5] Rasines I, Gómez M, Martín I, Rodríguez C, Mañanós E, Chereguini O. Artificial fertilization of Senegalese sole (*Solea senegal*ensis): Hormone therapy administration methods, timing of ovulation and viability of eggs retained in the ovarian cavity. Aquaculture. 2012;326–329: 129–135.

[6] Morais S, Aragão C, Cabrita E, Conceição LEC, Constenla M, Costas B, et al New developments and biological insights into the farming of *Solea senegalensis* reinforcing its aquaculture potential. Rev Aquac. 2016;8:227–263.

[7] Cabrita E, Anel L, Herraéz MP. Effect of external cryoprotectants as membrane stabilizers on cryopreserved rainbow trout sperm. Theriogenology. 2001;56: 623–35. 11572443 10.1016/s0093-691x(01)00594-5

[8] Cabrita E, Robles V, Rebordinos L, Sarasquete C, Herráez MP. Evaluation of DNA damage in rainbow trout (*Oncorhynchus mykiss*) and gilthead sea bream (*Sparus aurata*) cryopreserved sperm. Cryobiology. 2005;50: 144–153. doi: 10.1016/j.cryobiol.2004.12.003 15843004 10.1016/j.cryobiol.2004.12.003

[9] Valcarce DG, Herráez MP, Chereguini O, Rodríguez C, Robles V. Selection of nonapoptotic sperm by magnetic-activated cell sorting in Senegalese sole (*Solea senegalensis*). Theriogenology. 2016; doi: 10.1016/j.theriogenology.2016.04.010 27173958 10.1016/j.theriogenology.2016.04.010

[10] Cabrita E, Sarasquete C, Martínez-Páramo S, Robles V, Beirão J, Pérez-Cerezales S, et al Cryopreservation of fish sperm: Applications and perspectives. J Appl Ichthyol. 2010;26: 623–635.

[11] Fraser L, Strzeżek J. Is there a relationship between the chromatin status and DNA fragmentation of boar spermatozoa following freezing–thawing? Theriogenology. 2007;68: 248–257. doi: 10.1016/j.theriogenology.2007.05.001 17543381 10.1016/j.theriogenology.2007.05.001

[12] Labbe C, Martoriati A, Devaux A, Maisse G. Effect of sperm cryopreservation on sperm DNA stability and progeny development in rainbow trout. Mol Reprod Dev. 2001;60: 397–404. doi: 10.1002/mrd.1102 11599051 10.1002/mrd.1102

[13] Zilli L, Schiavone R, Zonno V, Storelli C, Vilella S. Evaluation of DNA damage in *Dicentrarchus labrax* sperm following cryopreservation. Cryobiology. 2003;47: 227–235. 14697734 10.1016/j.cryobiol.2003.10.002

[14] Dietrich GJ, Szpyrka A, Wojtczak M, Dobosz S, Goryczko K, Zakowski L, et al Effects of UV irradiation and hydrogen peroxide on DNA fragmentation, motility and fertilizing ability of rainbow trout (*Oncorhynchus mykiss*) spermatozoa. Theriogenology. 2005;64: 1809–22. doi: 10.1016/j.theriogenology.2005.04.010 15921734 10.1016/j.theriogenology.2005.04.010

[15] Beirão J, Cabrita E, Soares F, Herráez MP, Dinis MT. Cellular damage in spermatozoa from wild-captured *Solea senegalensis* as detected by two different assays: comet analysis and Annexin V-Fluorescein staining. J Appl Ichthyol. 2008;24: 508–513.

[16] Pérez-Cerezales S, Martínez-Páramo S, Cabrita E, Martínez-Pastor F, de Paz P, Herráez MP. Evaluation of oxidative DNA damage promoted by storage in sperm from sex-reversed rainbow trout. Theriogenology. 2009;71: 605–613. doi: 10.1016/j.theriogenology.2008.09.057 19117601 10.1016/j.theriogenology.2008.09.057

[17] Valcarce DG, Cartón-García F, Herráez MP, Robles V. Effect of cryopreservation on human sperm messenger RNAs crucial for fertilization and early embryo development. Cryobiology. 2013;67: 84–90. doi: 10.1016/j.cryobiol.2013.05.007 23727067 10.1016/j.cryobiol.2013.05.007

[18] Guerra SM, Valcarce DG, Cabrita E, Robles V. Analysis of transcripts in gilthead seabream sperm and zebrafish testicular cells: mRNA profile as a predictor of gamete quality. Aquaculture. 2013;406: 28–33.

[19] Roberts RJ, Agius C, Saliba C, Bossier P, Sung YY. Heat shock proteins (chaperones) in fish and shellfish and their potential role in relation to fish health: a review. J Fish Dis. 2010;33: 789–801. doi: 10.1111/j.1365-2761.2010.01183.x 20678104 10.1111/j.1365-2761.2010.01183.x

[20] Ferlin A, Speltra E, Patassini C, Pati MA, Garolla A, Caretta N, et al Heat Shock Protein and Heat Shock Factor Expression in Sperm: Relation to Oligozoospermia and Varicocele. J Urol. 2010;183: 1248–1252. doi: 10.1016/j.juro.2009.11.009 20096881 10.1016/j.juro.2009.11.009

[21] Li K, Xue Y, Chen A, Jiang Y, Xie H, Shi Q, et al Heat Shock Protein 90 Has Roles in Intracellular Calcium Homeostasis, Protein Tyrosine Phosphorylation Regulation, and Progesterone-Responsive Sperm Function in Human Sperm. Zhang M, editor. PLoS One. 2014;9: e115841 doi: 10.1371/journal.pone.0115841 25541943 10.1371/journal.pone.0115841 PMC4277372

[22] Matwee C, Kamaruddin M, Betts DH, Basrur PK, King WA. The effects of antibodies to heat shock protein 70 in fertilization and embryo development. Mol Hum Reprod. 2001;7: 829–37. 11517289 10.1093/molehr/7.9.829

[23] Neuer A, Spandorfer SD, Giraldo P, Jeremias J, Dieterle S, Korneeva I, et al Heat Shock Protein Expression During Gametogenesis and Embryogenesis. Infect Dis Obstet Gynecol Dis Obs Gynecol. 1999;7: 10–1610. 10.1155/S1064744999000034 PMC1784712 10231002

[24] Jodar M, Selvaraju S, Sendler E, Diamond MP, Krawetz SA. The presence, role and clinical use of spermatozoal RNAs for the Reproductive Medicine Network. Hum Reprod Update. 2013;19: 604–624. doi: 10.1093/humupd/dmt031 23856356 10.1093/humupd/dmt031 PMC3796946

[25] Meseguer M, de los Santos MJ, Simón C, Pellicer A, Remohí J, Garrido N. Effect of sperm glutathione peroxidases 1 and 4 on embryo asymmetry and blastocyst quality in oocyte donation cycles. Fertil Steril. 2006;86: 1376–1385. doi: 10.1016/j.fertnstert.2006.03.053 16979635 10.1016/j.fertnstert.2006.03.053

[26] Cabrita E, Soares F, Beirão J, García-López A, Martínez-Rodríguez G, Dinis MT. Endocrine and milt response of Senegalese sole, *Solea senegalensis*, males maintained in captivity. Theriogenology. 2011;75: 1–9. doi: 10.1016/j.theriogenology.2010.07.003 20833416 10.1016/j.theriogenology.2010.07.003

[27] Chereguini O, Garcia de la Banda I, Herrera M, Martinez C, De la Hera M. Cryopreservation of turbot *Scophthalmus maximus (L*.*)* sperm: fertilization and hatching rates. Aquac Res. 2003;34: 739–747.

[28] Cabrita E, Robles V, Rebordinos L, Sarasquete C, Herráez MP. Evaluation of DNA damage in rainbow trout (*Oncorhynchus mykiss*) and gilthead sea bream (*Sparus aurata*) cryopreserved sperm. Cryobiology. 2005;50: 144–53. doi: 10.1016/j.cryobiol.2004.12.003 15843004 10.1016/j.cryobiol.2004.12.003

[29] Martínez-Páramo S, Diogo P, Dinis MT, Herráez MP, Sarasquete C, Cabrita E. Incorporation of ascorbic acid and α-tocopherol to the extender media to enhance antioxidant system of cryopreserved sea bass sperm. Theriogenology. 2012;77: 1129–36. doi: 10.1016/j.theriogenology.2011.10.017 22153272 10.1016/j.theriogenology.2011.10.017

[30] Tapia-Paniagua ST, Vidal S, Lobo C, García de la Banda I, Esteban MA, Balebona MC, et al Dietary administration of the probiotic SpPdp11: Effects on the intestinal microbiota and immune-related gene expression of farmed *Solea senegalensis* treated with oxytetracycline. Fish Shellfish Immunol. 2015;46: 449–58. doi: 10.1016/j.fsi.2015.07.007 26190256 10.1016/j.fsi.2015.07.007

[31] Manchado M, Salas-Leiton E, Infante C, Ponce M, Asensio E, Crespo A, et al Molecular characterization, gene expression and transcriptional regulation of cytosolic HSP90 genes in the flatfish Senegalese sole (*Solea senegalensis Kaup*). Gene. 2008;416: 77–84. doi: 10.1016/j.gene.2008.03.007 18442885 10.1016/j.gene.2008.03.007

[32] Livak KJ, Schmittgen TD. Analysis of Relative Gene Expression Data Using Real-Time Quantitative PCR and the 2−^ΔΔCT^ Method. Methods. 2001;25: 402–408. doi: 10.1006/meth.2001.1262 11846609 10.1006/meth.2001.1262

[33] Infante C, Matsuoka MP, Asensio E, Canavate JP, Reith M, Manchado M. Selection of housekeeping genes for gene expression studies in larvae from flatfish using real-time PCR. BMC Mol Biol. 2008;9: 28 doi: 10.1186/1471-2199-9-28 18325098 10.1186/1471-2199-9-28 PMC2275743

[34] De Spiegelaere W, Dern-Wieloch J, Weigel R, Schumacher V, Schorle H, Nettersheim D, et al Reference gene validation for RT-qPCR, a note on different available software packages. PLoS One. 2015;10: e0122515 doi: 10.1371/journal.pone.0122515 25825906 10.1371/journal.pone.0122515 PMC4380439

[35] Yuan JS, Reed A, Chen F, Stewart CN. Statistical analysis of real-time PCR data. BMC Bioinformatics. 2006;7: 85 doi: 10.1186/1471-2105-7-85 16504059 10.1186/1471-2105-7-85 PMC1395339

[36] Robles V, Herráez P, Labbé C, Cabrita E, Pšenička M, Valcarce DG, et al Molecular basis of spermatogenesis and sperm quality. Gen Comp Endocrinol. 2016; doi: 10.1016/j.ygcen.2016.04.026 27131389 10.1016/j.ygcen.2016.04.026

[37] Li P, Li Z-H, Dzyuba B, Hulak M, Rodina M, Linhart O. Evaluating the impacts of osmotic and oxidative stress on common carp (*Cyprinus carpio*, *L*.) sperm caused by cryopreservation techniques. Biol Reprod. 2010;83: 852–8. doi: 10.1095/biolreprod.110.085852 20668258 10.1095/biolreprod.110.085852

[38] Cartón-García F, Riesco MF, Cabrita E, Herráez MP, Robles V. Quantification of lesions in nuclear and mitochondrial genes of *Sparus aurata* cryopreserved sperm. Aquaculture. 2013;402: 106–112.

[39] Halliwell B. Free radicals, antioxidants, and human disease: curiosity, cause, or consequence? Lancet. Elsevier; 1994;344: 721–724. 10.1016/s0140-6736(94)92211-x 7915779

[40] Walter CA, Intano GW, McCarrey JR, McMahan CA, Walter RB. Mutation frequency declines during spermatogenesis in young mice but increases in old mice. Proc Natl Acad Sci. 1998;95: 10015–9. 9707592 10.1073/pnas.95.17.10015 PMC21453

[41] Zilli L, Schiavone R, Zonno V, Storelli C, Vilella S. Evaluation of DNA damage in *Dicentrarchus labrax* sperm following cryopreservation. Cryobiology. 2003;47: 227–35. 14697734 10.1016/j.cryobiol.2003.10.002

[42] Hagedorn M, McCarthy M, Carter VL, Meyers SA, Knight J, Abbot A, et al Oxidative Stress in Zebrafish (*Danio rerio*) Sperm. PLoS One. Public. 2012;7: e39397. 10.1371/journal.pone.0039397 PMC3378538 22724013

[43] Gray DC, Mahrus S, Wells JA. Activation of specific apoptotic caspases with an engineered small-molecule-activated protease. Cell. 2010;142: 637–46. doi: 10.1016/j.cell.2010.07.014 20723762 10.1016/j.cell.2010.07.014 PMC3689538

[44] Martin G, Sabido O, Durand P, Levy R. Cryopreservation induces an apoptosis-like mechanism in bull sperm. Biol Reprod. 2004 71: 28–37. doi: 10.1095/biolreprod.103.024281 14973261 10.1095/biolreprod.103.024281

[45] Said TM, Agarwal A, Zborowski M, Grunewald S, Glander H-J, Paasch U. Utility of magnetic cell separation as a molecular sperm preparation technique. J. Androl. 2008;29, 134–42. doi: 10.2164/jandrol.107.003632 18077822 10.2164/jandrol.107.003632 PMC2801545

[46] Riesco MF, Robles V. Cryopreservation Causes Genetic and Epigenetic Changes in Zebrafish Genital Ridges. PLoS One. 2013;8: e67614 doi: 10.1371/journal.pone.0067614 23805321 10.1371/journal.pone.0067614 PMC3689738

[47] Meseguer M, Garrido N, Simón C, Pellicer a, Remohí J. Concentration of glutathione and expression of glutathione peroxidases 1 and 4 in fresh sperm provide a forecast of the outcome of cryopreservation of human spermatozoa. J Androl. 2004;25: 773–80. 15292110 10.1002/j.1939-4640.2004.tb02855.x

[48] Steger K. Haploid spermatids exhibit translationally repressed mRNAs. Anat Embryol (Berl). 2001;203: 323–34. 11411307 10.1007/s004290100176

[49] Robles V, Herráez P, Labbé C, Cabrita E, Pšenička M, Valcarce DG, et al Molecular basis of spermatogenesis and sperm quality. Gen Comp Endocrinol. 2016; doi: 10.1016/j.ygcen.2016.04.026 27131389 10.1016/j.ygcen.2016.04.026

[50] Ostermeier GC, Dix DJ, Miller D, Khatri P, Krawetz SA, Bhasin S, et al Spermatozoal RNA profiles of normal fertile men. Lancet. 2002;360: 772–777. doi: 10.1016/S0140-6736(02)09899-9 12241836 10.1016/S0140-6736(02)09899-9

[51] Valcarce DG, Cartón-García F, Riesco MF, Herráez MP, Robles V. Analysis of DNA damage after human sperm cryopreservation in genes crucial for fertilization and early embryo development. Andrology. 2013;1: 723–730. doi: 10.1111/j.2047-2927.2013.00116.x 23970451 10.1111/j.2047-2927.2013.00116.x

[52] González-Rojo S, Fernández-Díez C, Guerra SM, Robles V, Herraez MP. Differential gene susceptibility to sperm DNA damage: analysis of developmental key genes in trout. PLoS One. 2014;9: e114161 doi: 10.1371/journal.pone.0114161 25479606 10.1371/journal.pone.0114161 PMC4257556

[53] Riesco MF, Robles V. Quantification of DNA damage by q-PCR in cryopreserved zebrafish Primordial Germ Cells. J Appl Ichthyol. 2012;28: 925–929.

[54] Agarwal A, Aponte-Mellado A, Premkumar BJ, Shaman A, Gupta S. The effects of oxidative stress on female reproduction: a review. Reprod Biol Endocrinol. BioMed Central. 2012;10: 49. 10.1186/1477-7827-10-49 PMC3527168 22748101

[55] Mehaisen GMK, Saeed AM, Gad A, Abass AO, Arafa M, El-Sayed A, et al Antioxidant Capacity of Melatonin on Preimplantation Development of Fresh and Vitrified Rabbit Embryos: Morphological and Molecular Aspects. PLoS One. 2015;10: e0139814 doi: 10.1371/journal.pone.0139814 26439391 10.1371/journal.pone.0139814 PMC4595475

[56] Yavaş İ, Bozkurt Y, Yıldız C. Cryopreservation of scaly carp (*Cyprinus carpio*) sperm: effect of different cryoprotectant concentrations on post-thaw motility, fertilization and hatching success of embryos. Aquac Int. Springer Netherlands. 2014;22: 141–148.

[57] Scott AP, Baynes SM. A review of the biology, handling and storage of salmonid spermatozoa. J Fish Biol. Blackwell Publishing Ltd; 1980;17: 707–739.

[58] Quinn PJ, Chow PY, White IG. Evidence that phospholipid protects ram spermatozoa from cold shock at a plasma membrane site. J Reprod Fertil. 1980;60: 403–7. 7431346 10.1530/jrf.0.0600403

[59] Alderson R, Macneil AJ. Preliminary investigations of cryopreservation of milt of Atlantic salmon (*Salmo salar*) and its application to commercial farming. Aquaculture. 1984;43: 351–354.

[60] Tekin N, Secer S, Akcay E, Bozkurt Y. Cryopreservation of Rainbow trout (*Oncorhynchus mykiss*) semen. Isr J Aquac–Bamidgeh. 2003;55: 208–212.

[61] Betsy CJ, Kumar JSS. Influence of Egg Yolk on the Quality of Cryopreserved Spermatozoa of Common Carp, *Cyprinus carpio*. J Appl Aquac. 2015;27: 40–49.

[62] Babiak I, Glogowski J, Luczynski MJ, Luczynski M, Demianowicz W. The effect of egg yolk, low density lipoproteins, methylxanthines and fertilization diluent on cryopreservation efficiency of northern pike (*Esox lucius*) spermatozoa. Theriogenology. Elsevier; 1999;52: 473–479. 10.1016/S0093-691X(99)00144-2 10734381

[63] Babiak I, Glogowski J, Kujawa R, Kucharczyk D, Mamcarz A. Cryopreservation of Sperm from Asp *Aspius aspius*. Progress Fish-Culturist. 1998;60: 146–148.

