# Supplementary figures and images for: Solea senegalensis sperm cryopreservation: New insights on sperm quality
Source: PLoS One. 2017 Oct 20;12(10):e0186542. doi: 10.1371/journal.pone.0186542 (PMC5650144; doi:10.1371/journal.pone.0186542)

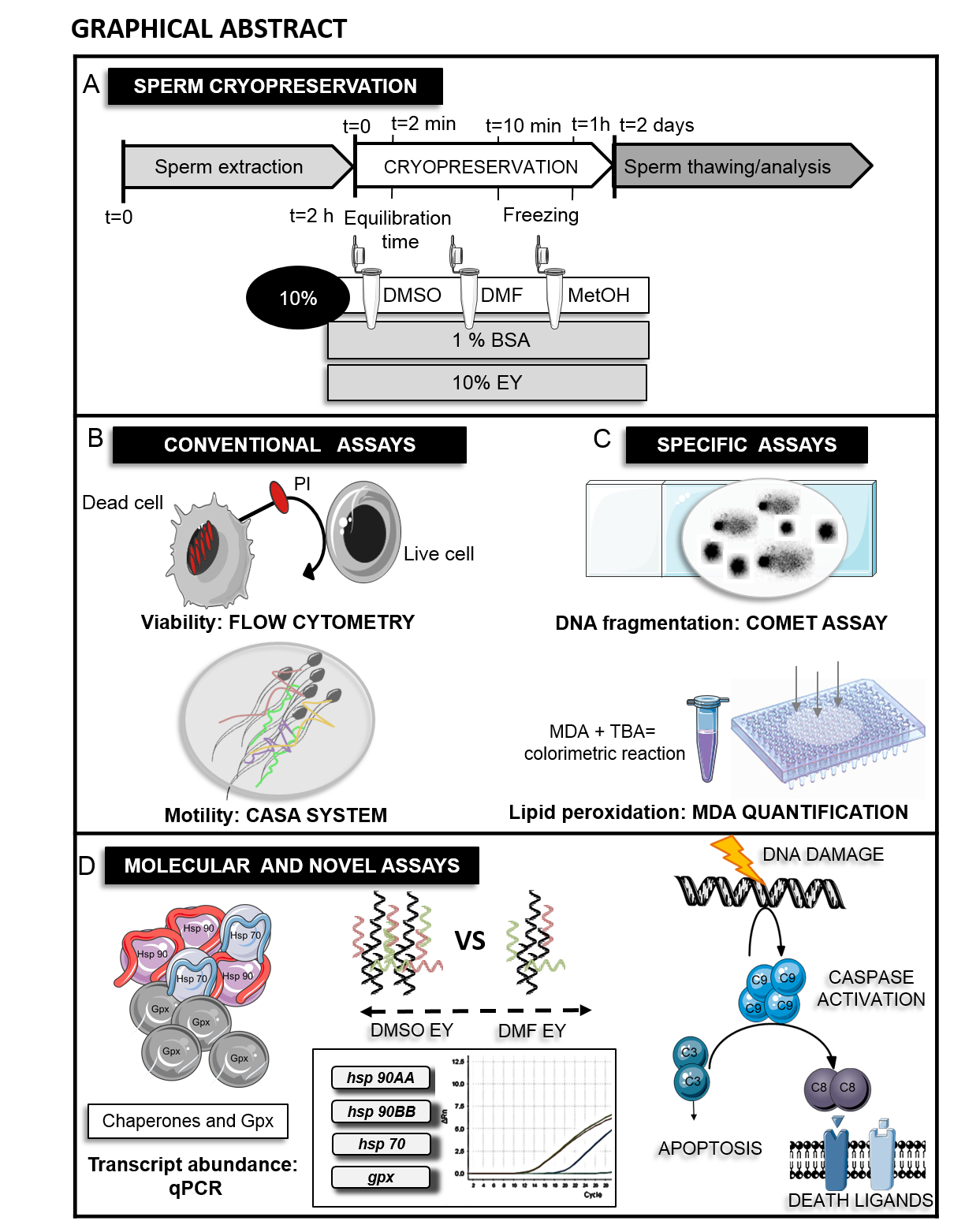

Supplement: S1 Fig — A) Sperm cryopreservation protocol, B) Conventional assays, C) Specific assays, D) Novel and molecular assays. (TIF) [file pone.0186542.s001.tif]

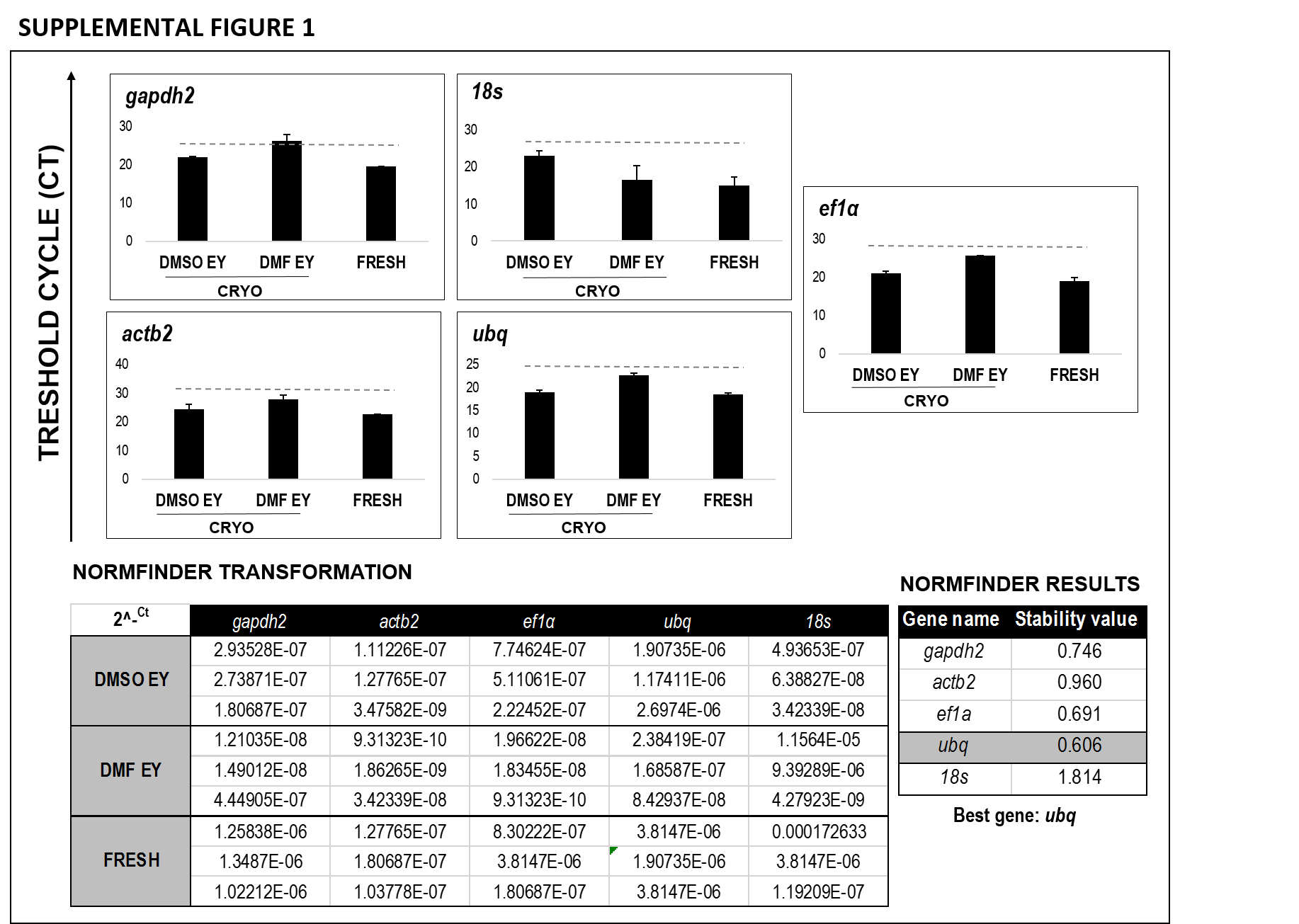

Supplement: S2 Fig — Different housekeeping genes were selected as glyceraldehyde-3-phosphate dehydrogenase (gapdh2), β-actin (actb2) or 18S ribosomal (18s) RNA, ubiquitin (ubq), and subunit a1 of elongation factor 1 gene, (ef1α). The Ct value indicates the fractional cycle at which fluorescence intensity reached the fluorescence threshold. For this analysis the best CPA conditions were analysed: dimethyl sulfoxide (DMSO) and dimethyl formamide (DMF) supplemented with 10% egg yolk (EY), compared to fresh control. Transcriptional levels (Ct values) of candidate reference genes were expressed as a mean ± SE (n = 3, and 3 technique replicates). The stability value was calculated after a 2^-Ct transformation using Normfinder software. (TIF) [file pone.0186542.s002.tif]

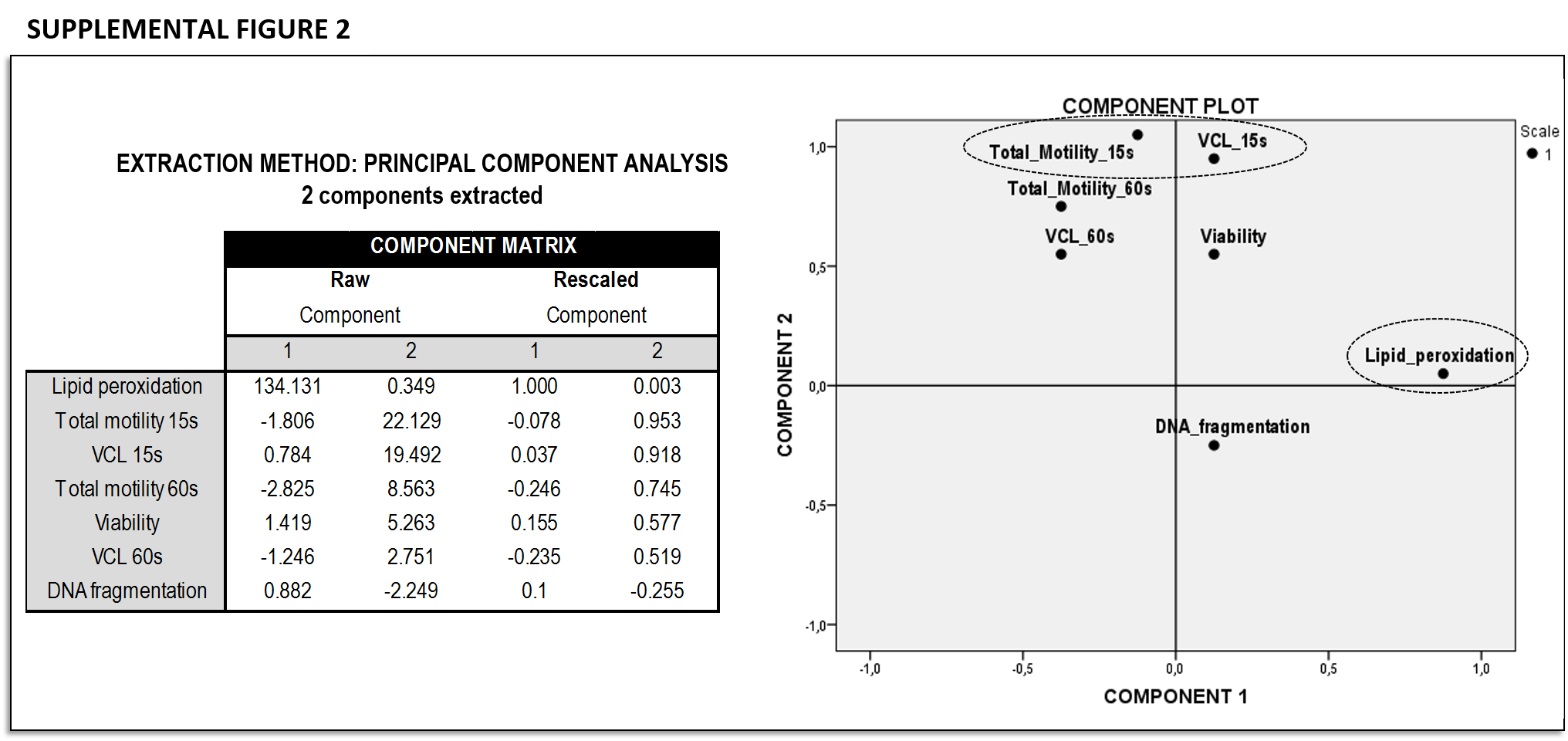

Supplement: S3 Fig — A Principal Component Analysis was performed to reduce the dimensionality of a data set. PCA results defined two components, one explaining 93% of the total variance of our results (component 1), and the other explaining 5% (component 2). Component 1 corresponds to lipid peroxidation data, and component 2 corresponds to motility parameters. (TIF) [file pone.0186542.s003.tif]
